# Gastrointestinal Fibroblasts Have Specialized, Diverse Transcriptional Phenotypes: A Comprehensive Gene Expression Analysis of Human Fibroblasts

**DOI:** 10.1371/journal.pone.0129241

**Published:** 2015-06-05

**Authors:** Youichi Higuchi, Motohiro Kojima, Genichiro Ishii, Kazuhiko Aoyagi, Hiroki Sasaki, Atsushi Ochiai

**Affiliations:** 1 Laboratory of Cancer Biology, Department of Integrated Biosciences, Graduate School of Frontier Sciences, The University of Tokyo, Kashiwa, Chiba, Japan; 2 Pathology Division, Research Center for Innovative Oncology National Cancer Center Hospital East, Kashiwa, Chiba, Japan; 3 Genetic Division, National Cancer Center Research Institute, Chuo-ku, Tokyo, Japan; INSERM, FRANCE

## Abstract

**Background:**

Fibroblasts are the principal stromal cells that exist in whole organs and play vital roles in many biological processes. Although the functional diversity of fibroblasts has been estimated, a comprehensive analysis of fibroblasts from the whole body has not been performed and their transcriptional diversity has not been sufficiently explored. The aim of this study was to elucidate the transcriptional diversity of human fibroblasts within the whole body.

**Methods:**

Global gene expression analysis was performed on 63 human primary fibroblasts from 13 organs. Of these, 32 fibroblasts from gastrointestinal organs (gastrointestinal fibroblasts: GIFs) were obtained from a pair of 2 anatomical sites: the submucosal layer (submucosal fibroblasts: SMFs) and the subperitoneal layer (subperitoneal fibroblasts: SPFs). Using hierarchical clustering analysis, we elucidated identifiable subgroups of fibroblasts and analyzed the transcriptional character of each subgroup.

**Results:**

In unsupervised clustering, 2 major clusters that separate GIFs and non-GIFs were observed. Organ- and anatomical site-dependent clusters within GIFs were also observed. The signature genes that discriminated GIFs from non-GIFs, SMFs from SPFs, and the fibroblasts of one organ from another organ consisted of genes associated with transcriptional regulation, signaling ligands, and extracellular matrix remodeling.

**Conclusions:**

GIFs are characteristic fibroblasts with specific gene expressions from transcriptional regulation, signaling ligands, and extracellular matrix remodeling related genes. In addition, the anatomical site- and organ-dependent diversity of GIFs was also discovered. These features of GIFs contribute to their specific physiological function and homeostatic maintenance, and create a functional diversity of the gastrointestinal tract.

## Introduction

Fibroblasts are cells with spindle-shaped morphology that reside in human connective tissue. They are the principal type of stromal cell and perform multiple physiological and pathological functions. In addition to their basic function of contributing to the maintenance of a structural framework and of tissue homeostasis with their ability to synthesize many extracellular matrix and growth factors, they also play an important role in fetal development, wound healing, and cancer progression [1, 2]. They are widely distributed within the body and play variable roles within organs with different functions. However, their lack of specific molecular markers and common morphological features hinder functional classification [3]. Despite this, in many organs, the topographical or anatomical diversity of fibroblasts has been investigated. In colonic tissue, subepithelial fibroblasts are known to contribute to the maintenance of epithelial cell homeostasis, wound healing, and immune responses [4, 5], and subperitoneal fibroblasts are known to produce peritoneal fluid and facilitate appropriate functioning of intra-abdominal organs [6, 7]. With that said, previous studies analyzing the diversity of fibroblasts have analyzed fibroblasts from only one or few organs, and comprehensive analyses examining the diversity of fibroblasts within the whole body are lacking. Recently, the global gene expressions of skin and colonic tissue fibroblasts have been reported, and their topological and anatomical diversities were elucidated [8–11]. Global gene expression analysis can be an effective and robust method to grasp the diversity of fibroblasts in an organ. Studies utilizing this method may determine site-specific markers of fibroblasts and generate basic data to understand the organ-specific microenvironments they create.

In this study, we obtained 63 human primary fibroblasts from 13 organs from around the whole body, and global gene expression analyses were performed using Affymetrix GeneChip U133 Plus 2. The aim of this study was to elucidate the transcriptional diversity of fibroblasts across the whole body and to reveal gene expression patterns that discriminate their diversity. To this end, we identified gastrointestinal fibroblasts (GIFs) as a group within the body with a special gene expression. Then, we analyzed the gene expression pattern of GIFs intensively to elucidate their transcriptional character. Further diversity within GIFs was also analyzed, and the genes that contributed to form diversity within GIFs were identified.

## Materials and Methods

### Ethics Statement

This study was approved by the National Cancer Center Hospital East Institutional Reviews Board (No: 19–021). A written comprehensive prior consent arrangement to use any biologic materials for research was obtained from each donor, including use of tissue sections, RNA, DNA, protein, and cultured cells. In this study, human primary fibroblasts (submucosal fibroblasts, SMFs; subperitoneal fibroblasts, SPFs; Lung tissue fibroblasts, LuFs; vascular adventitial fibroblasts, VAFs; breast dermal fibroblasts, DeFs; mammary fibroblasts from Japanese subjects, J_MaFs; liver fibroblasts, LiFs; and gallbladder fibroblasts, GaFs), tissue total RNA samples, and tissue section samples were obtained with protocols approved by the institutional review board.

### Isolation and primary culture of fibroblasts

Human primary fibroblasts were obtained from surgically resected normal tissues more than 5 cm away from the tumor. GIFs were obtained from the esophagus, pyloric antrum of the stomach, third portion of the duodenum, terminal ileum, and sigmoid colon. These tissues were separated into submucosal and subperitoneal tissue as described previously [11, 12]. Gastrointestinal tissue was dissected from the muscular layer on the luminal side, and the lamina propria and mucosal layer tissues were obtained. Next, the lamina propria was scrubbed away to obtain submucosal tissue. Subperitoneal tissue was obtained from the mesentery of gastrointestinal tissue by using operating tweezers and scissors (S1A Fig). After being washed with phosphate buffered saline (PBS) 3 times, each tissue was incubated in PBS with 0.05% trypsin (Sigma) for 4 hours at 37°C. After incubation, the remaining tissue was rejected, and the trypsin solution was centrifuged to obtain a pellet of fibroblast cells. Then cells were seeded into 6 cm dishes (BD Falcon) and cultured at 37°C and 5.0% CO_2_ condition. GaFs were obtained from the gallbladder wall using trypsin incubation. LuFs, VAFs, DeFs, J_MaFs, and LiFs were obtained by attaching the tissue sections to the plastic dish as described previously [13]. Tissues were washed with PBS, minced into small pieces with a diameter of approximately 5 mm, attached on a 6 cm dish with 1.0 mL of MF medium (Toyobo), and cultured at 37°C and 5.0% CO_2_ condition. After migrated spindle-shaped cells were observed, tissue pieces were removed to obtain fibroblasts. Purchased cell lines were 3 mammary fibroblasts from Caucasians (C_MaFs; Zenbio), 2 hepatic stellate cells (HSCs; Zenbio), 3 uterine fibroblasts (UtFs; LIFE LINE CELL TECHNOLOGY), and 3 prostate fibroblasts (PrFs; ScienCell). All fibroblasts were grown and maintained in MF medium and characterization using immunofluorescence staining and flow cytometry analysis was performed to check their culture purity (S1B and S1C Fig). All experiments were performed with cells within 8 passages. Patient information for each fibroblast, including the donor’s sex, age, and race, are described in S1 Table.

### Immunofluorescence staining

For the immunofluorescence staining of fibroblasts, 2.0×10^3^ fibroblasts were plated on a culture slide (BD Falcon) and cultured for 2 days. Cultured cells were fixed in cold methanol for 10 minutes and cold acetone for 5 minutes on ice. After fixation, cells were blocked in 2% NSS/PBS for 30 minutes. Cells were incubated with primary antibodies at room temperature for 1 hour. After PBS washing, cells were incubated with goat anti-mouse Alexa Flour 488 nm (Invitrogen), goat anti-rabbit Alexa Flour 488 nm (Invitrogen), or donkey anti-goat Alexa Flour 546 nm at room temperature for 1 hour, and then mounted with VectaShield Mounting Medium with DAPI (Vector Laboratories, Inc.) for counterstaining. For the confirmation of the expression of msh homeobox 1 (*MSX1*) in human gastrointestinal tissue, paraffin-embedded tissue was obtained from 3 surgically resected human normal colonic and gastric tissues. After deparaffinization of the tissue, antigen retrieval was performed with pH 9.0 Tris-EDTA buffer (Dako) at 95°C for 20 minutes, and the tissue was incubated with mouse-monoclonal anti-Vimentin (Dako) and rabbit-polyclonal anti-MSX1 (Sigma) at 4°C overnight followed by goat anti-mouse Alexa Flour 546 nm (Invitrogen) and goat anti-rabbit Alexa Flour 488 nm. Ten fields of submucosal and subserosal areas were captured and the nuclear protein expressions of *MSX1* in vimentin-positive, spindle-shaped fibroblastic cells were evaluated by calculating the color difference [14]. The immunofluorescence images were captured on an Axio Imager M1 (Zeiss) with AxioCam HRc (Zeiss), and images were analyzed with Axio Vision 4.7.1 (Zeiss) and Photoshop CS5 (Adobe Systems). The primary antibodies used are described in S2 Table.

### Flow cytometry analysis

Flow cytometry analysis was performed to characterize the cell surface antigen of the fibroblasts. FACSCalibur (Beckton-Dickinson, San Jose, CA) was used and a minimum of 10,000 events counted with Cell Quest software (Beckton-Dickinson Labware, Franklin Lakes, NF). Cells were trypsinized, centrifuged, and incubated with primary antibody for 15 minutes on ice in a dark condition. They were then washed with PBS containing 3% fetal bovine serum (FBS; Sigma) and 0.05% Na_2_N_3_, and then were incubated with rabbit anti-mouse IgG/FITC (Dako) as a secondary antibody for 15 minutes on ice in a dark condition. Washing with PBS again, a FACS scan was performed using Cell Quest software. The primary antibodies used are described in S2 Table.

### Isolation and purification of total RNA

To obtain the total RNA from cultured fibroblasts, 5.0×10^5^ fibroblasts were plated on a 10 cm dish (BD Falcon) and cultured in Dulbecco’s modified Eagle's medium (DMEM; Sigma) with 10% FBS and 1% penicillin streptomycin (Sigma) for 48 hours. Then, the medium was changed into DMEM without FBS and the cells were cultured for 48 hours. After culturing, the cells were washed with PBS and suspended in 1.0 mL of TRIzol reagent (Invitrogen) using Cell Scraper (SARSTEDT), and stored at -80°C. The total RNA was purified from thawed samples using TRIzol / RNeasy minicolumn protocol (QIAGEN). RNase-free DNase (QIAGEN) was treated on column for 15 minutes to remove the minimum genomic DNA contamination. A quality check of all total RNA samples was performed using Agilent Bioanalyzer with an RNA 6000 Nano Assay kit (Agilent Technology), and confirmed that the RNA integrity numbers of all RNA samples were > 9.0.

To obtain the total RNA from human tissue, we homogenized the separated submucosal and subperitoneal tissues into TRIzol reagent using Tissue Lyser II (QIAGEN), and stored the solution at -80°C. Purification of total RNA was performed as described earlier.

### Gene expression analysis using microarrays

We used GeneChip Human Genome U133 Plus 2 arrays (Affymetrix), containing 54,675 probe sets, to analyze the mRNA expression levels of approximately 47,000 transcripts and variants from 38,500 well-characterized human genes. Target complementary RNA was generated from 100 ng of total RNA from each sample using a 3’ IVT Express Kit (Affymetrix). The procedures for target hybridization, washing, and staining with signal amplification were conducted according to the supplier's protocols. The arrays were scanned with a GeneChip Scanner 3000 (Affymetrix). The primary expression microarray data are available at Gene Expression Omnibus (http://www.ncbi.nlm.nih.gov/geo/; NCBI). The accession number of 63 primary fibroblasts microarray data is GSE63626; of these, 3 pairs of colon SMFs and SPFs microarray data are previously reported in GSE53059.

### Statistical analysis of microarrays

Gene expression data were analyzed with GeneSpring GX12.6 (Agilent Technologies). Raw data were summarized using microarray suite 5 (MAS5) algorithm and normalized into log transformed and median centered data to perform the numerical analysis to permit gene selection.

For unsupervised hierarchical clustering, we used probe sets that were reliably measured and varied by 3-fold above the global median in at least 10% of samples. The differentially expressed probe sets used in supervised hierarchical clustering were selected based on *P* < 0.05 and fold change > 2.0. *P* values were calculated using unpaired *t*-test or one way ANOVA with Benjamini and Hochberg multiple correction. For hierarchical clustering, average linkage clustering with Pearson correlation distance was performed.

### Validation of GIF signature genes in independent microarray data sets

To validate the distinct gene expression between GIFs and non-GIFs, we used the public microarray data of human primary fibroblasts analyzed using Affymetrix GeneChip U133A Plus 2 array. Three data sets of colon mucosal fibroblasts (GSE15322, GSE29316, GSE39394) and 1 data set of stomach fibroblasts (GSE44740) were used as GIF samples, and 2 data sets of mammary gland fibroblasts (GSE20086, GSE25619) and 2 data sets of lung fibroblasts (GSE23066, GSE44723) were used as non-GIF samples [15–22]. Hierarchical clustering based on 995 GIF signature genes were performed, and whether the first branch distinguishes the GIFs and non-GIFs or not were validated.

### Quantitative real-time reverse transcriptase-polymerase chain reaction (qRT-PCR)

The cDNA was synthesized using the PrimScript RT reagent Kit (TaKaRa), according to the manufacturer’s protocol. The qRT-PCR was performed in a Light Cycler System (Roche) using SYBR Premix Ex Taq (Tli RNaseH Plus; TaKaRa) according to the manufacturer’s protocol. The target gene expression was normalized with the gene expression of *GAPDH*. The primer designs used in this study are described in S3 Table.

### Statistical analysis

**Fig 1:**
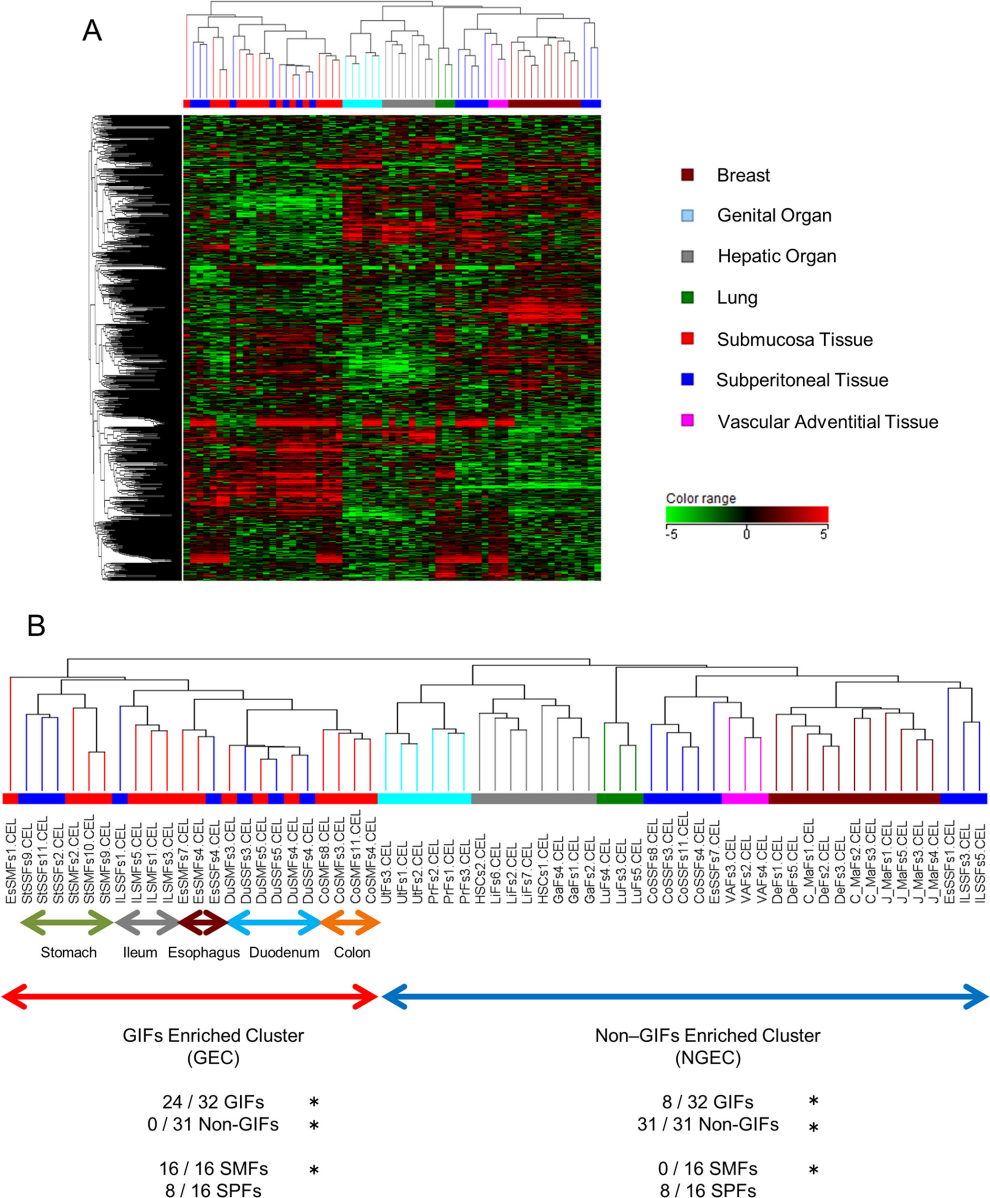
Diversity of Gene Expression in Human Gastrointestinal Fibroblasts. (A) Diversity of gene expression of 63 primary human fibroblasts. Each column is colored with the fibroblast origin; dermal fibroblasts (DeFs), mammary fibroblasts from Japanese (J_MaFs), mammary fibroblasts from Caucasians (C_MaFs) are colored brown; prostate fibroblasts (PrFs) and uterus fibroblasts (UtFs) are colored light blue; gallbladder fibroblasts (GaFs), hepatic stellate cells (HSCs), and liver fibroblasts (LiFs) are colored gray; lung fibroblasts (LuFs) are colored green; submucosal fibroblasts from the gastrointestinal tract (SMFs) are colored red; subperitoneal fibroblasts from the gastrointestinal tract (SPFs) are colored blue; and vascular adventitial fibroblasts (VAFs) are colored pink. The color scale of the gene expression range is +5 to -5 logs on log base 2. (B) Expansion view of the dendrogram seen in (A). The first bifurcation of the dendrogram separated gastrointestinal fibroblasts (GIFs) and non-GIFs significantly (*P* < 0.001). Furthermore, clustering depending on anatomical site and gastrointestinal organ was also observed.

The significance of differences between any 2 groups was evaluated by using Student’s *t*-test. A difference was considered significant at *P* < 0.05. The significance of distribution of GIFs or non-GIFs, SMFs, and SPFs in Fig 1 were evaluated by the χ^2^ test. The error bars show the mean ± SEM.

## Results

### GIFs show a characteristic and diverse gene expression profile depending on their organ and anatomical site

To elucidate the transcriptional diversity of fibroblasts, 63 samples of human primary fibroblasts from the whole body, including 13 organs with anatomical variability, were cultured. All obtained cells were easily adhered to the plastic dish, grew well with at least 6 passages, and showed spindle-shaped morphology (S1A Fig). Immunofluorescence staining and flow cytometry analysis revealed that 11 of 13 obtained cells were positive for mesenchymal marker (vimentin, CD105), but negative for epithelial marker (cytokeratin AE1/3), smooth muscle marker (desmin), neural cells marker (GFAP), mesothelial marker (calretinin), endothelial marker (CD31), hematopoietic cells marker (CD34), lymphocyte marker (CD45), and monocyte marker (CD68; S1B and S1C Fig). We successfully confirmed almost all obtained cells were fibroblasts. Expectably, GaFs and LiFs were positive for desmin, which is the characteristic phenotype of stellate cells, like stromal cells in hepatic organs [23, 24].

The global gene expression profiles of the fibroblasts were analyzed using Affymetrix GeneChip U133 Plus 2. To characterize the transcriptional diversity of fibroblasts, 776 probe sets that were reliably measured and whose expression varied at least 3-fold from the median across all samples in at least 10% of samples were selected, and unsupervised hierarchical clustering analysis was performed. Overall, the first bifurcation of the dendrogram separated fibroblasts into 2 subgroups: GIFs enriched cluster (GEC) and non–GIFs enriched cluster (NGEC). Twenty-four of 32 GIFs were included in GEC, and 32 of 32 non-GIFs were in NGEC (*P* < 0.001, χ^2^ test). All of the 8 GIFs in NGEC were SPFs, whereas all SMFs were in GEC (*P* < 0.001). These results suggested the transcriptional specialty of GIFs. Organ-dependent clusters were also found both in GIFs and non-GIFs. However, fibroblasts were not clustered by the donor’s age or sex (Fig 1A and 1B, S1 Table). Further, HSCs (Zenbio) from Caucasians were located in the same cluster with LiFs (fibroblasts from liver) from Japanese donors, and C_MaFs (Zenbio) from Caucasians were located in the same cluster with J_MaFs and DeFs from Japanese donors (fibroblasts from mammary gland and breast dermal). Therefore, the transcriptional diversity of fibroblasts was not explained by the racial difference. Further, SMFs and SPFs in the colon, ileum, and stomach formed independent clusters, whereas those in duodenum and esophagus did not. These results indicated the existence of transcriptional diversity between SMFs and SPFs in some gastrointestinal organs.

### The different transcriptional character between GIFs and non-GIFs

**Fig 2:**
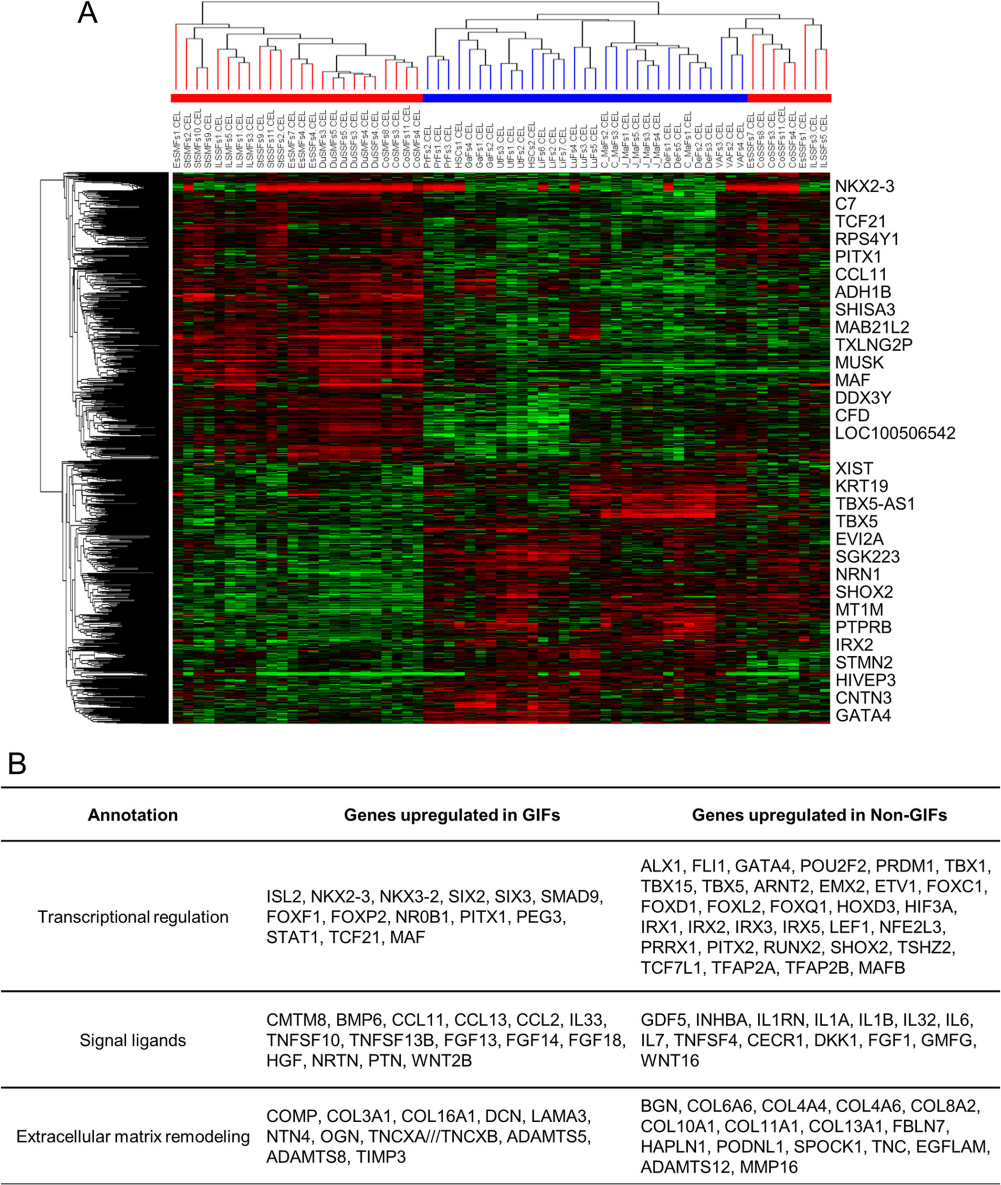
The Transcriptional Differences between Gastrointestinal Fibroblasts and Non–Gastrointestinal Fibroblasts. (A) Supervised clustering using the significant different expressed probe sets between gastrointestinal fibroblasts (GIFs) and non-GIFs. The 995 significant probe sets were selected based on *P* < 0.05 and fold change > 2.0 (one-way ANOVA). The red bars indicate a GIF sample, and blue bars indicate a non-GIF sample. The top significant genes are shown on the right. The color scale of gene expression is the same as Fig 1A. (B) Distinct expression of the genes related with transcriptional regulation, signal ligands, and extracellular matrix remodeling in GIFs and Non-GIFs.

Next, we selected the significant different expressing probe sets between GIFs and non-GIFs, and a supervised hierarchical clustering analysis was performed. We selected 995 probe sets of GIF signature genes based on *P* < 0.05 (unpaired *t*-test) and fold change > 2.0 (Fig 2A and S4 Table). The GIF signature genes consisted of many genes related with transcriptional regulation (transcriptional factors and co-factors), signal ligands (growth factors, cytokine, chemokine and other hormonal factors), and extracellular matrix regeneration (collagen molecules, proteoglycans, matrix metalloproteinase: Fig 2B). In addition, the validation study of these signature genes was performed using 8 independent microarray data sets of human primary fibroblasts in public GEO datasets, including 3 sets of colon mucosal fibroblasts (GSE15322, GSE29316, GSE39394), 1 set of stomach mucosal fibroblasts (GSE44740), 2 sets of normal mammary gland fibroblasts (GSE20086, GSE25619), and 2 sets of lung fibroblasts (GSE23066, GSE44723; lung tissue and airway fibroblasts). By hierarchical cluster analysis using 995 GIF signature genes, we successfully determined that the first bifurcation of the dendrogram separated 11 GIF samples and 18 non-GIF samples into different clusters (S2A–S2C Fig). These results revealed that the differences between GIFs and non–GIFs were characterized by their expression of genes related to transcriptional regulation, signal ligands, and extracellular matrix remodeling.

### Anatomical site dependent diversity in GIFs

**Fig 3:**
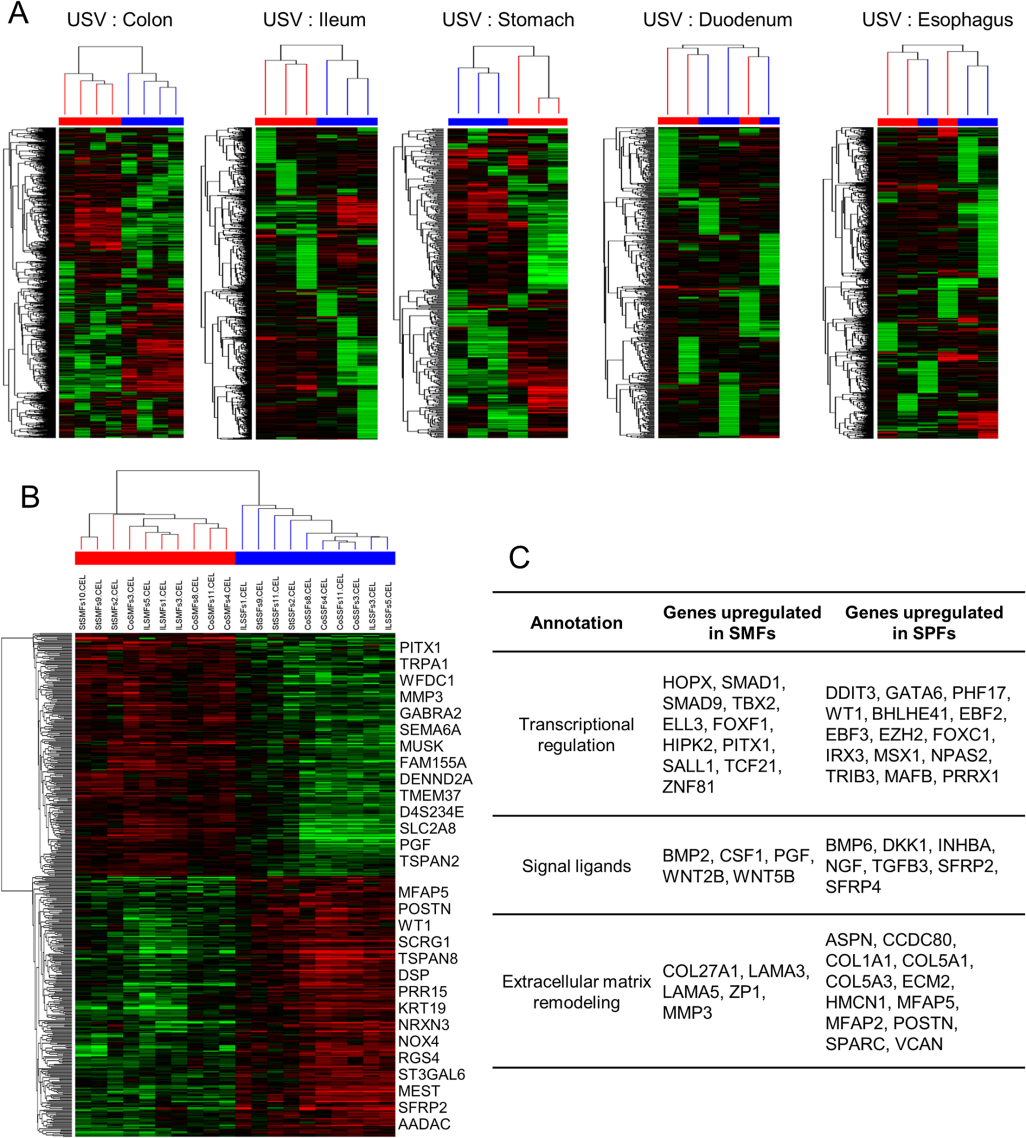
Anatomical Site Dependent Diversity in Gastrointestinal Fibroblasts. (A) Unsupervised clustering in the colon, ileum, stomach, duodenum, and esophagus derived fibroblasts samples. Submucosal fibroblasts (SMFs) and subperitoneal fibroblasts (SPFs) from the colon, ileum, and stomach showed different gene expression profiles, whereas those from the duodenum and esophagus were not separated into different clusters. The red bar indicates SMFs samples and the blue bar indicates SPFs samples. The color scale of gene expression is same as Fig 1A. (B) Supervised clustering between SMFs and SPFs samples in the colon, ileum, and stomach. A total of 498 probe sets were selected based on *P* < 0.05 and fold change > 2.0 (*t*-test unpaired). The top significant genes are shown on the right. (C) Distinct expression of the genes related with transcriptional regulation, signal ligands, and extracellular matrix remodeling in SMFs and SPFs.

To elucidate the transcriptional difference between different anatomical sites of SMFs and SPFs, unsupervised clustering was performed in each organ within the gastrointestinal tract. Although we found a transcriptional difference between SMFs and SPFs in the colon, ileum, and stomach, these differences were not found in the duodenum and esophagus (Fig 3A). Therefore, supervised analyses between SMFs and SPFs were performed for stomach, ileum, and colon fibroblasts, and 498 probe sets were identified as anatomical site signature genes in GIFs (Fig 3B and S5 Table). The anatomical site signature genes were also characterized with the genes related with transcriptional regulation, signal ligands, and extracellular matrix remodeling (Fig 3C).

### Organ dependent diversity in GIFs

**Fig 4:**
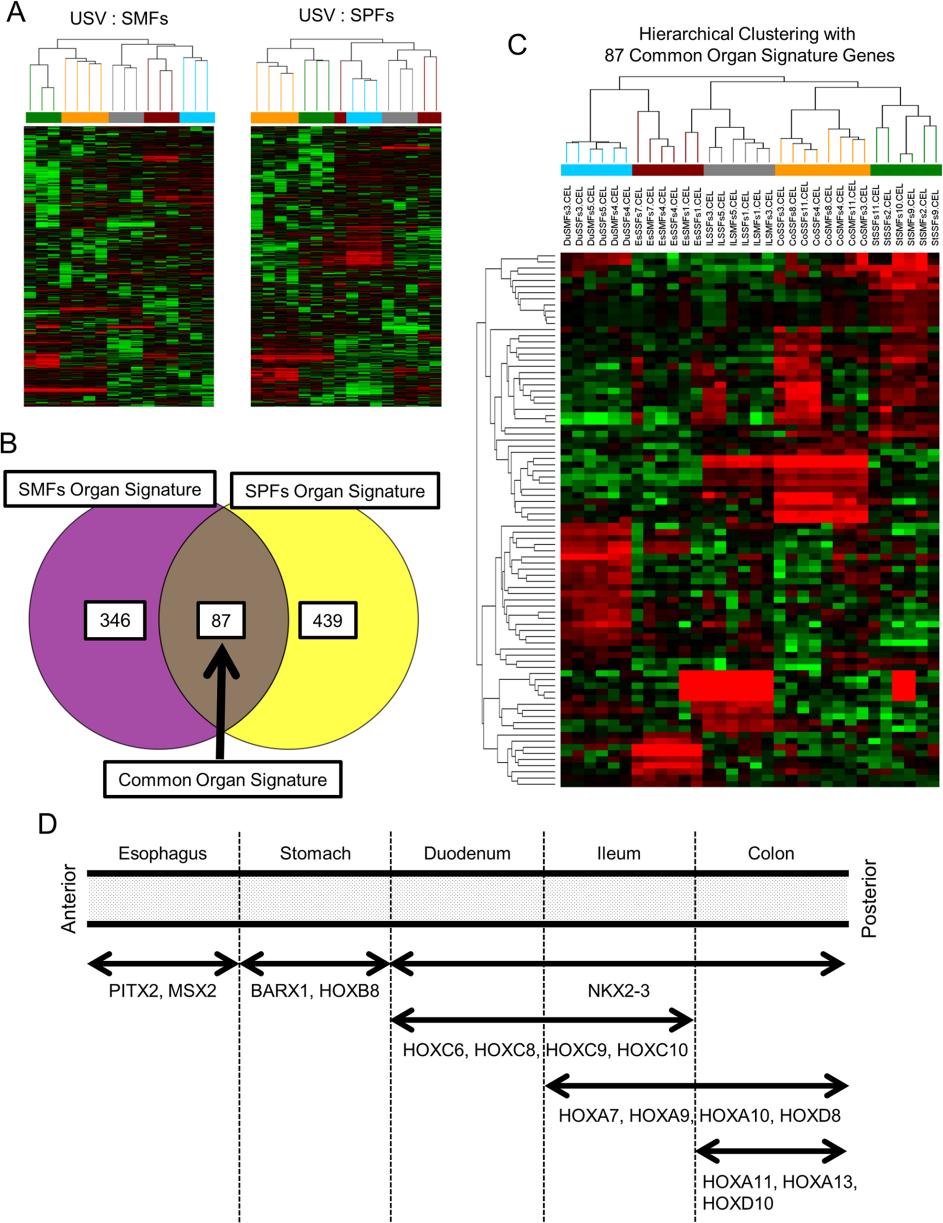
Organ Dependent Diversity in Gastrointestinal Fibroblasts. (A) Unsupervised clustering within submucosal fibroblasts (SMFs) or subperitoneal fibroblasts (SPFs) samples. In both analyses, samples tended to be separated with their organs. The bar indicates each gastrointestinal organ: esophagus (brown), stomach (green), duodenum (light blue), ileum (gray), and colon (orange). (B) Organ signature genes in SMFs and SPFs. The probe sets that express specifically in each organ were selected based on *P* < 0.05, fold change > 2.0. The 433 probe sets were selected as SMFs organ signature genes, and 526 probe sets were selected as SPFs organ signature genes; of these, 87 probe sets expressed both SMFs and SPFs, defined as common organ signature genes. (C) Hierarchical clustering based on 87 common organ signature genes. The samples were clustered with their organs, but not with their anatomical site. (D) A schematic image of the expression pattern of homeotic genes in GIFs. The regional expression of homeotic genes depending on the anterior-posterior axis of the gastrointestinal tract was observed.

Next, we investigated the organ-dependent diversity in GIFs. Because the diversity of GIFs was also influenced by their anatomical site, unsupervised analysis was performed in SMFs and SPFs separately. Unsupervised analysis revealed that both SMFs and SPFs had an organ-dependent diversity (Fig 4A). Next, based on *P* < 0.05 (one way ANOVA) and fold change > 2.0, the organ-specific genes in SMFs and SPFs were selected respectively. Then using a Venn diagram of organ specific genes in SMFs and SPFs, we identified 87 probe sets of common organ signature genes (Fig 4B and S6 Table). Hierarchical clustering based on these 87 probe sets successfully drew the dendrogram that separated GIF samples into their organs, but not into their anatomical sites (Fig 4C). Because many homeotic genes were observed in common organ signature genes (15 of 87 probe sets), unsupervised clustering based on 35 probe sets of homeotic genes that were relatively expressed in GIFs was performed. Interestingly, these homeotic genes tended to separate GIF samples into their organs, and showed regional expression patterns depending on the anterior-posterior axis of gastrointestinal tract. (Fig 4D and S3 Fig).

### Anatomical site signature genes and common organ signature genes discriminate the topological diversity of GIFs

**Fig 5:**
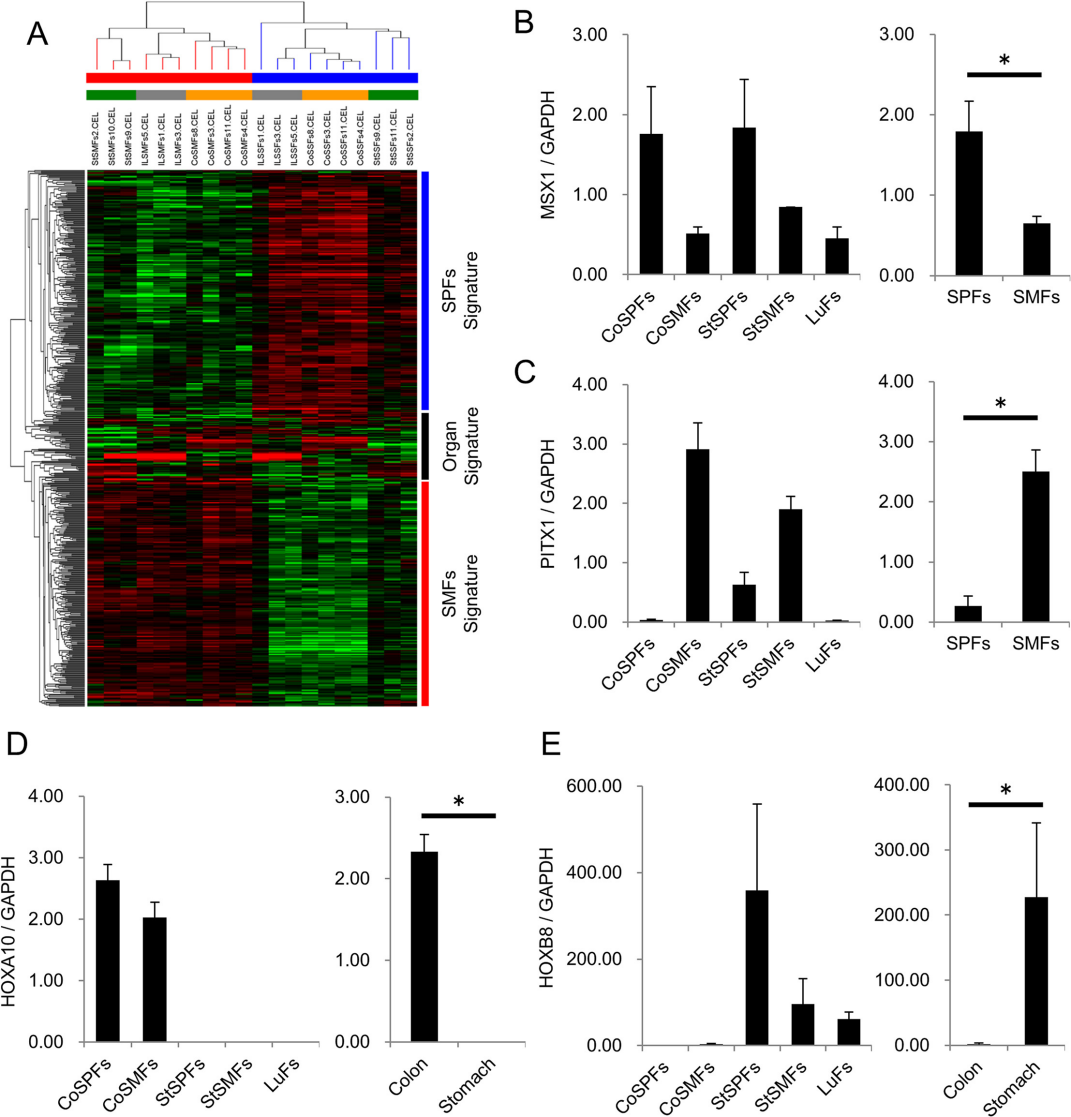
Anatomical Site and Organ Signature Genes Discriminate the Topological Diversity of Gastrointestinal Fibroblasts. (A) Hierarchical clustering of the stomach, ileum, and colon fibroblasts, based on 585 probe sets that consisted of 498 anatomical site signature genes and 87 common organ signature genes, as shown in Figs 3 and 4. The bar indicates the positional information of the samples: upper bar indicates submucosal fibroblasts (red) or subperitoneal fibroblasts (blue), and the lower bar indicates stomach (gray), ileum (green), or colon (orange). (B-E) The validation study of the anatomical site and organ signature genes in independent fibroblasts samples. The mRNA expression of SPFs signature: *MSX1* (B), SMFs signature: *PITX1* (C), colon fibroblasts signature: *HOXA10* (D), and stomach fibroblasts signature: *HOXB8* (E) were calculated (*n* = 3).

We found that the diversity of stomach, ileum, and colon fibroblasts is explained by the anatomical site signature genes and common organ signature genes. To test the distinction ability of these signature genes, we united 87 probe sets of common organ signature genes and 498 probe sets of anatomical site signature genes, and performed hierarchical clustering. As a result, the dendrogram showed the cluster that separated the samples depended on both their anatomical site and organ (Fig 5A). Using qRT-PCR and immunofluorescence staining, we confirmed the mRNA and protein expression of the signature genes, paired-like homeodomain 1 (*PITX1)* in SMFs, *MSX1* in SPFs, homeobox A10 (*HOXA10*) in colon fibroblasts, and homeobox B8 (*HOXB8*) in stomach fibroblasts in independent samples (Fig 5B–5E, S4 Fig and S5 Fig). These results indicate that the transcriptional diversity within GIFs was mainly discriminated by their 2 topological axes, which are anatomical site and gastrointestinal organ.

### Expression of anatomical site signature genes and organ signature genes in human gastrointestinal tissue

**Fig 6:**
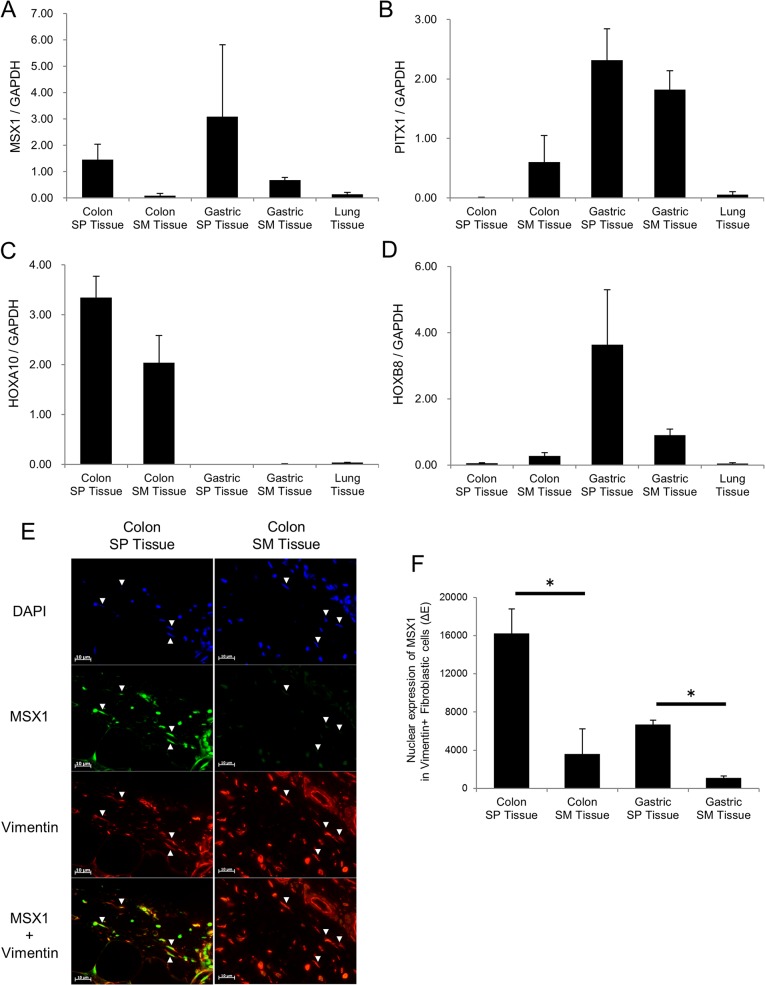
The Expression of Anatomical Site and Organ Signature Genes in Human Tissue. (A-D) The mRNA expression of anatomical and organ signature genes in human mesenchymal tissue (*n* = 3). SPFs signature: *MSX1* (A), SMFs signature: *PITX1* (B), colon fibroblasts signature: *HOXA10* (C), stomach fibroblasts signature: *HOXB8* (D). (E) Immunofluorescence staining of *MSX1* in human colonic tissue. Arrow heads show vimentin-positive, spindle-shaped fibroblastic cells. (F) Semi-quantitative value of nuclear *MSX1* expression in human colonic and gastric tissue (*n* = 3).

Finally, we confirmed the expression of anatomical site and organ signature genes in human colonic and gastric mesenchymal tissue. Using qRT-PCR, we confirmed that the gene expression of *MSX1*, *HOXA10*, and *HOXB8* in human mesenchymal tissue was correlated with their expression *in vitro*, whereas we failed to confirm the gene expression of *PITX1* (Fig 6A–6D). Furthermore, using immunofluorescence staining, we observed the protein expression of SPFs signature gene *MSX1* in human tissue. We calculated the nuclear protein expression of *MSX1* in vimentin-positive, spindle-shaped fibroblastic cells, and confirmed that SPFs showed higher expression of *MSX1* than SMFs *in vivo* (Fig 6E and 6F, S6 Fig).

## Discussion

In this study, using fibroblasts from various organs, we demonstrated a detailed gene expression pattern of fibroblasts within the whole body. We firstly elucidated the origin-dependent transcriptional diversity of fibroblasts from the whole body, and found a distinct gene expression pattern in GIFs. Furthermore, we found anatomical site and organ dependent diversity in GIFs. The anatomical site and organ dependent diversity within GIFs were explained by the expression of the genes related with transcriptional regulation, signal ligands, and extracellular matrix remodeling. Our data of transcriptome analysis of human fibroblasts is the widest systematic study to provide direct evidence indicating specialized, diverse transcriptional phenotypes of GIFs.

One of the vital roles of fibroblasts is secreting extracellular matrix to provide a structural framework and maintain homeostasis in tissue [25]. In this study, we observed the distinct gene expression of some collagen molecules, microfibrils, glycoproteins, proteoglycans, and matrix metalloproteinase in fibroblasts with various origins. Owing to various, site-specific expressions of these extracellular matrix genes, GIFs may create a tissue-specific mechanical microenvironment to support the physical function of gastrointestinal organs.

Another vital role of fibroblasts is to regionalize the other cell types, such as epithelial cells, into tissue-specific phenotypes via embryonic development through reciprocal epithelial and mesenchymal interaction. Although epithelial cells are the major cell type that contributes to organ-specific physiological function in the gastrointestinal tract, their regionalization depending on the anterior-posterior axis is organized by mesenchymal cells [26–29]. Our data on GIFs with distinct transcriptional factors that relate to the developmental process validate that expression can be associated with epithelial regionalization and consequently allow GIFs to play distinct and variable physiological functions within gastrointestinal organs [30–32]. These site-specific gene expressions in fibroblasts seemed to be involved not only in the developmental process, but also in the tissue-specific differentiation of other cell types, including epithelial cells or mast cells [33–35]. Therefore, the elucidated organ-dependent diversity of GIFs may also be involved in the tissue-specific differentiation of adult stem cells in the gastrointestinal tract.

Site-specific expression of humoral signal ligands, which include growth factors, cytokine and chemokine, and some Wnt, BMP, or TGF-beta signaling ligands, were also elucidated. In gastrointestinal tissue, such signal ligands contribute to homeostasis maintenance by supporting epithelial cell and smooth muscle cell proliferation and by attracting immune cells [36]. Many previous reports suggest that GIFs secrete various kinds of humoral factors and can create a cocktail to support tissue homeostasis [37–39]. Submucosal compartment of gastrointestinal tract exist between two distinct smooth muscle layers of the muscularis mucosae and muscularis propria. And the characteristic humoral factor expression of SMFs may contribute to the homeostasis of these smooth muscle tissues. Further, in specific conditions, such as a wound repairing process, some recent studies suggest that mesenchymal cells in the peritoneal area migrate to the wounded area of the epithelium, and contribute to the proliferation and structural morphogenesis of intestinal epithelium via non-canonical Wnt signaling [40–42]. Taken together with our observation of distinct expressions of humoral signaling factors, fibroblasts in each anatomical site may perform independent physiological functions, and potentially contribute to tissue homeostasis by distinct signaling pathways.

Although all SMFs showed typical GIF transcriptional phenotypes, 8 of 16 SPFs showed non-GIF like transcriptional phenotypes. The human peritoneum is comprised of 2 anatomically distinct areas: the visceral and the parietal peritoneum. Whereas gastrointestinal organs are largely invested by the visceral peritoneum, some other organs, including the esophageal adventitia or duodenum peritoneum, are invested by parietal peritoneum. Furthermore, peritoneal tissue is histologically variable. For example, gastric peritoneum shows a fat-less thin histologic appearance, whereas colonic peritoneum shows a thick histologic appearance with abundant fat tissue. Such anatomical and histological heterogeneity of peritoneum tissue may contribute to the transcriptional diversity of SPFs. Next, interestingly, SPFs with non-GIF like phenotype showed transcriptional similarity with vascular adventitial fibroblasts (VAFs). Anatomically, vascular adventitia and serosa have continuity in the lung, and vascular adventitia and serosal tissue are known to contain a prominent elastic fiber component [43]. Previously, we reported both VAFs and SPFs possess robust tumor progression ability, and our results suggest the existence of a fibroblastic subgroup with special pathological function. Therefore, our data can potentially provide not only basic data about physiological function, but also important clues to estimate pathological processes of fibroblasts.

In conclusion, GIFs are a distinct subgroup within the whole body, and were subclassified depending on their anatomical site or organ. These heterogeneous transcriptional phenotypes were mainly discriminated by the expression pattern of the genes related to transcriptional regulation, humoral signaling ligands, and extracellular matrix remodeling. The site-specific phenotypes of fibroblasts are related to embryogenesis, and may contribute to create the organ- or site-specific microenvironment necessary to maintain tissue homeostasis. These new data further demonstrate the wide spectrum of physiological and pathological roles these cells can play, and can be an important resource for future organogenetic studies.

## Supporting Information

S1 Fig Isolation and Characterization of Human Primary Fibroblasts. (A) Schema of isolating human submucosal and subperitoneal fibroblasts. Human gastrointestinal tissue was separated into submucosal tissue and subperitoneal tissue, and fibroblasts were isolated from each tissue in pairs. (B) Characterization of human primary fibroblasts with immunofluorescence staining. Vimentin: mesenchymal marker; Cytokeratin: epithelial marker; Desmin: smooth muscle marker; GFAP: neural cells marker. (C) Characterization of cell surface antigens of human primary fibroblasts. CD31: endothelial marker; CD34: hematopoietic marker; CD45: lymphocyte marker; CD68: monocyte marker; CD105: mesenchymal marker.
(TIF)

S2 Fig Validation study of 995 Gastrointestinal Fibroblast Signature Genes in Public GEO Datasets. **(Related to Fig 2).** (A) Hierarchical clustering of public human primary fibroblasts microarray data sets using 995 GIFs signature genes. An orange bar indicates colon fibroblasts samples (GIFs), a gray bar indicates stomach fibroblasts samples (GIFs), a brown bar indicates mammary gland fibroblasts samples (non-GIFs), and a green bar indicates lung fibroblasts samples (non-GIFs). The first branch of the dendrogram separated samples into GIF samples and non-GIF samples. (B, C) The expression of the GIF signature gene in validation data. GIF specific gene: *PITX1* (B) and non-GIFs specific gene: *TBX5* (C) are shown. (D, E) The expression of organ signature genes of GIFs in validation data. Colon fibroblasts specific gene: *HOXA13* (D) and stomach fibroblasts specific gene: *BARX1* (E) are shown.
(TIF)

S3 Fig Diverse Expression of Homeotic Genes in Gastrointestinal Fibroblasts. **(Related to Fig 4).** Unsupervised hierarchical clustering of GIFs based on 35 probe sets of homeotic genes that were relatively expressed. The bar indicates each gastrointestinal organ: esophagus (brown), stomach (green), duodenum (blue), ileum (gray), and colon (red).
(TIF)

S4 Fig Protein expression of Anatomical Site Signature Genes in Gastrointestinal Fibroblasts. **(Related with Fig 5).** (A) Immunofluorescence imaging of SPFs signature gene: *MSX1* in colon SMFs and SPFs. Arrow heads indicating the fibroblasts with nuclear staining of *MSX1*. (B) Quantification of the ratio of *MSX1* positive cells in colon and stomach SMFs and SPFs (*n* = 3). (C) Immunofluorescence imaging of SMFs signature gene: *PITX1* in colon SMFs and SPFs. (D) Quantification of the ratio of *PITX1* positive cells in colon and stomach SMFs and SPFs (*n* = 3).
(TIF)

S5 Fig Protein expression of Organ Signature Genes in Gastrointestinal Fibroblasts. **(Related with Fig 5).** (A) Immunofluorescence imaging of colon fibroblasts signature gene: *HOXA10* in colon and stomach SMFs. Arrow heads indicating the fibroblasts with nuclear staining of *HOXA10*. (B) Quantification of the ratio of *HOXA10* positive cells in colon and stomach SMFs and SPFs (*n* = 3). (C) Immunofluorescence imaging of stomach fibroblasts signature gene: *HOXB8* in colon and stomach SMFs. (D) Quantification of the ratio of *HOXB8* positive cells in colon and stomach SMFs and SPFs (*n* = 3).
(TIF)

S6 Fig Determination of the Exposure Times to Semi-Quantitate the Nuclear Expression of *MSX1* in Human Gastrointestinal Tissue. **(Related to Fig 6).** (A) Immunofluorescence image of *MSX1* in colon subperitoneal tissue for each exposure time. The fluorescence image of *MSX1* shows clear and bright at an exposure time of 8.0 ms, without any background staining. Arrow heads indicate the vimentin-positive, spindle-shaped fibroblastic cells in the picture. (B) Immunofluorescence image of *MSX1* in colon submucosal tissue for each exposure time. A weak fluorescence image can be observed at exposure time 8.0 ms. Arrow heads indicate the vimentin-positive, spindle-shaped fibroblastic cells in the picture. (C) Semi-quantitation of the nuclear expression of *MSX1* in human gastrointestinal tissue fibroblasts. Nuclear color difference of the vimentin-positive, spindle-shaped fibroblastic cells in each picture were measured. The error bars show the mean ± SD of 4 fibroblastic cells in the picture.
(TIF)

S1 Table Specimen information of the primary fibroblasts used for microarray analysis. (XLSX)

S2 Table Primary antibodies used in this study. (XLSX)

S3 Table Primer sequences used for qRT-PCR analysis. (XLSX)

S4 Table List of 995 probe sets of gastrointestinal fibroblast signature genes. (XLSX)

S5 Table List of 498 probe sets of anatomical site signature genes. (XLSX)

S6 Table List of 87 probe sets of common organ signature genes. (XLSX)
