# Supplementary figures and images for: Gastrointestinal Fibroblasts Have Specialized, Diverse Transcriptional Phenotypes: A Comprehensive Gene Expression Analysis of Human Fibroblasts
Source: PLoS One. 2015 Jun 5;10(6):e0129241. doi: 10.1371/journal.pone.0129241 (PMC4457624; doi:10.1371/journal.pone.0129241)

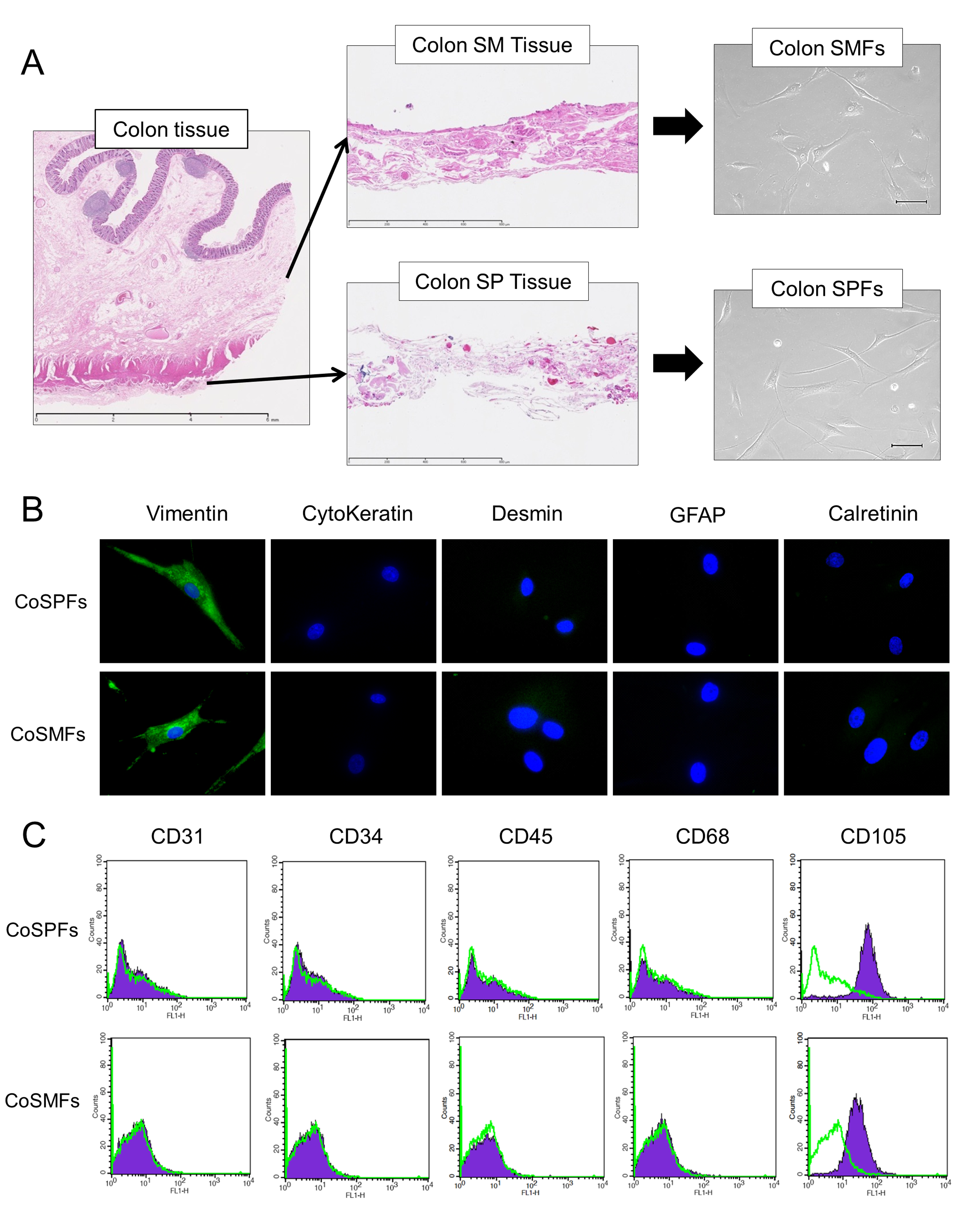

Supplement: S1 Fig — (A) Schema of isolating human submucosal and subperitoneal fibroblasts. Human gastrointestinal tissue was separated into submucosal tissue and subperitoneal tissue, and fibroblasts were isolated from each tissue in pairs. (B) Characterization of human primary fibroblasts with immunofluorescence staining. Vimentin: mesenchymal marker; Cytokeratin: epithelial marker; Desmin: smooth muscle marker; GFAP: neural cells marker. (C) Characterization of cell surface antigens of human primary fibroblasts. CD31: endothelial marker; CD34: hematopoietic marker; CD45: lymphocyte marker; CD68: monocyte marker; CD105: mesenchymal marker. (TIF) [file pone.0129241.s001.tif]

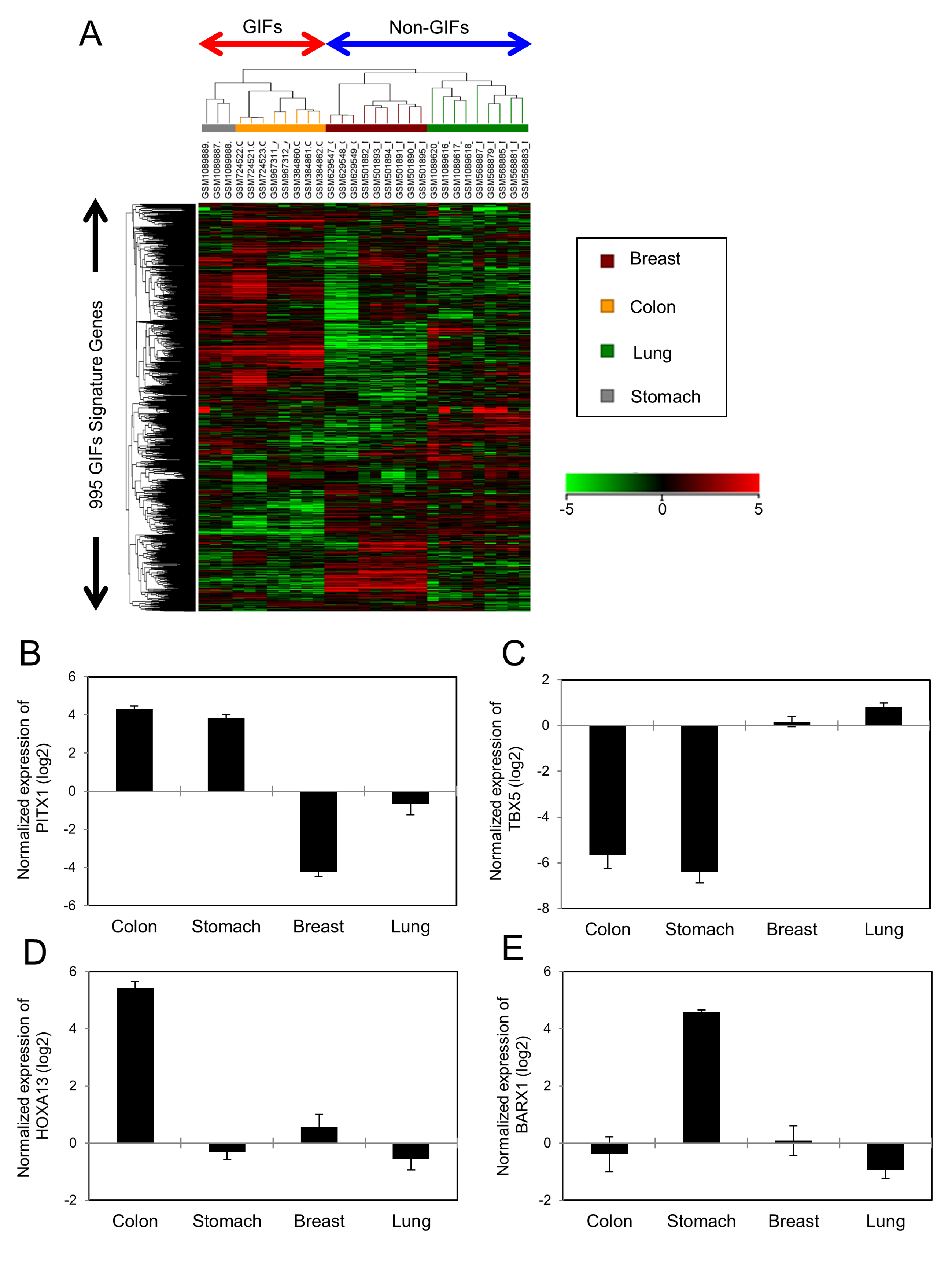

Supplement: S2 Fig — (Related to Fig 2). (A) Hierarchical clustering of public human primary fibroblasts microarray data sets using 995 GIFs signature genes. An orange bar indicates colon fibroblasts samples (GIFs), a gray bar indicates stomach fibroblasts samples (GIFs), a brown bar indicates mammary gland fibroblasts samples (non-GIFs), and a green bar indicates lung fibroblasts samples (non-GIFs). The first branch of the dendrogram separated samples into GIF samples and non-GIF samples. (B, C) The expression of the GIF signature gene in validation data. GIF specific gene: PITX1 (B) and non-GIFs specific gene: TBX5 (C) are shown. (D, E) The expression of organ signature genes of GIFs in validation data. Colon fibroblasts specific gene: HOXA13 (D) and stomach fibroblasts specific gene: BARX1 (E) are shown. (TIF) [file pone.0129241.s002.tif]

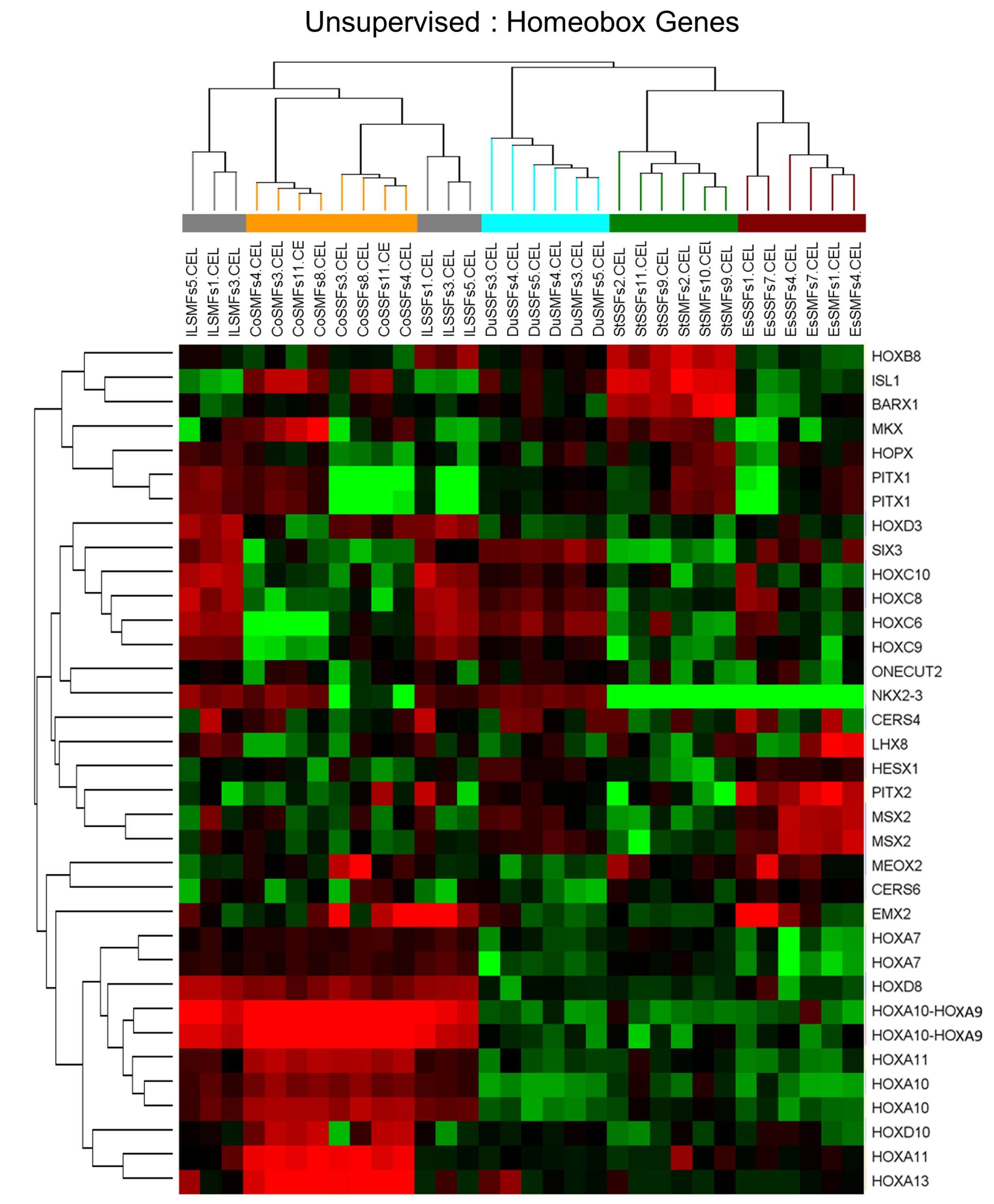

Supplement: S3 Fig — (Related to Fig 4). Unsupervised hierarchical clustering of GIFs based on 35 probe sets of homeotic genes that were relatively expressed. The bar indicates each gastrointestinal organ: esophagus (brown), stomach (green), duodenum (blue), ileum (gray), and colon (red). (TIF) [file pone.0129241.s003.tif]

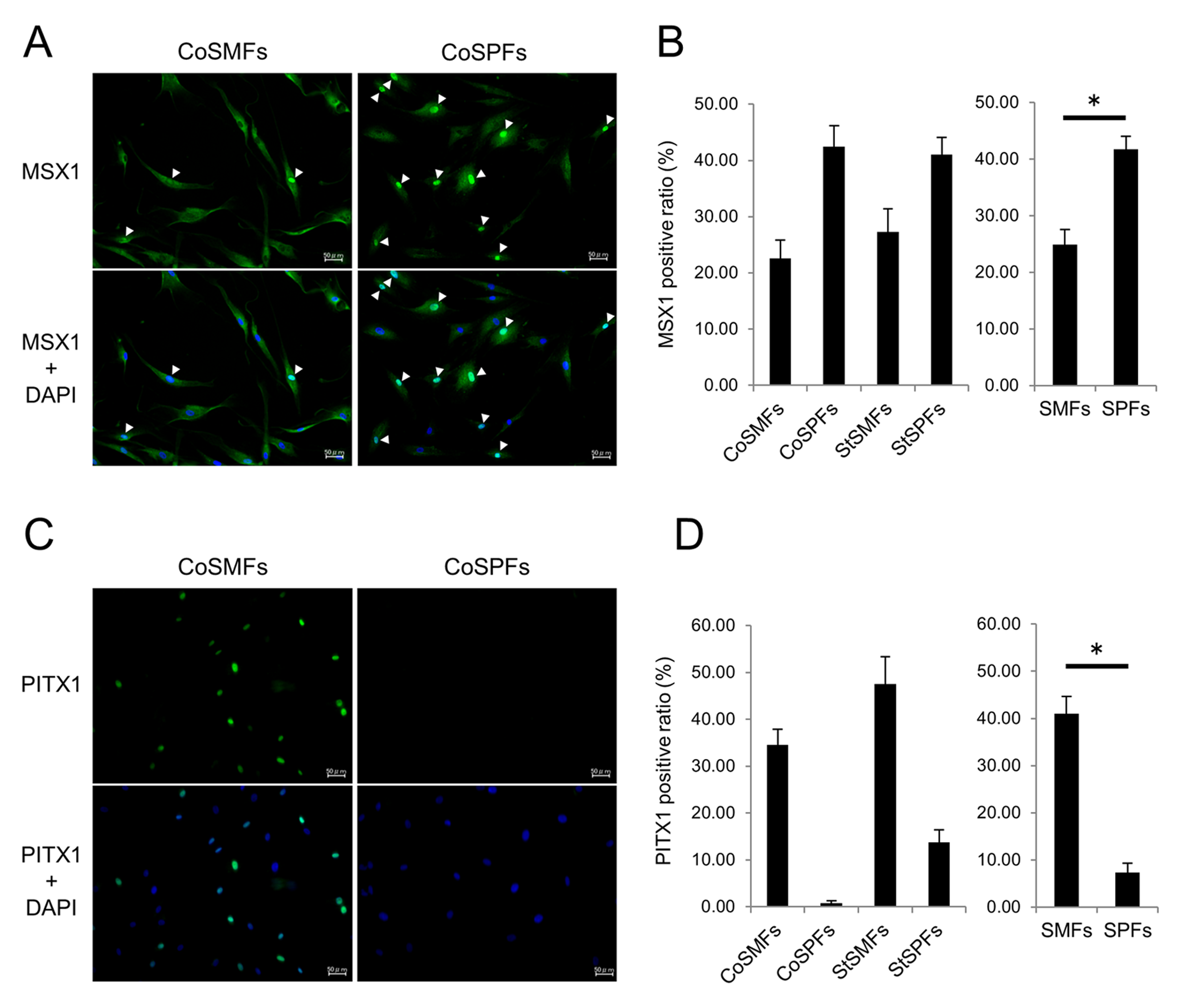

Supplement: S4 Fig — (Related with Fig 5). (A) Immunofluorescence imaging of SPFs signature gene: MSX1 in colon SMFs and SPFs. Arrow heads indicating the fibroblasts with nuclear staining of MSX1. (B) Quantification of the ratio of MSX1 positive cells in colon and stomach SMFs and SPFs (n = 3). (C) Immunofluorescence imaging of SMFs signature gene: PITX1 in colon SMFs and SPFs. (D) Quantification of the ratio of PITX1 positive cells in colon and stomach SMFs and SPFs (n = 3). (TIF) [file pone.0129241.s004.tif]

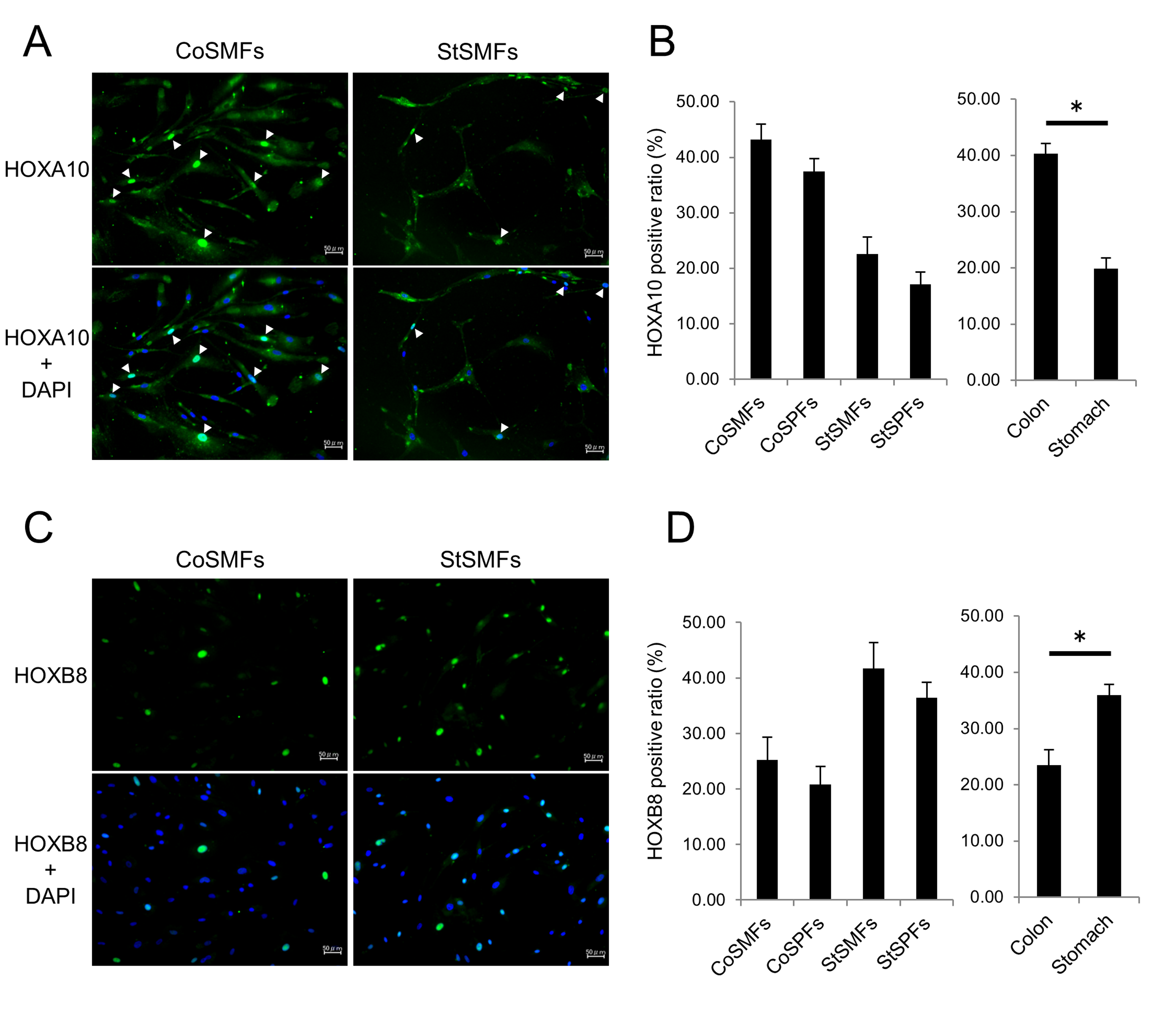

Supplement: S5 Fig — (Related with Fig 5). (A) Immunofluorescence imaging of colon fibroblasts signature gene: HOXA10 in colon and stomach SMFs. Arrow heads indicating the fibroblasts with nuclear staining of HOXA10. (B) Quantification of the ratio of HOXA10 positive cells in colon and stomach SMFs and SPFs (n = 3). (C) Immunofluorescence imaging of stomach fibroblasts signature gene: HOXB8 in colon and stomach SMFs. (D) Quantification of the ratio of HOXB8 positive cells in colon and stomach SMFs and SPFs (n = 3). (TIF) [file pone.0129241.s005.tif]

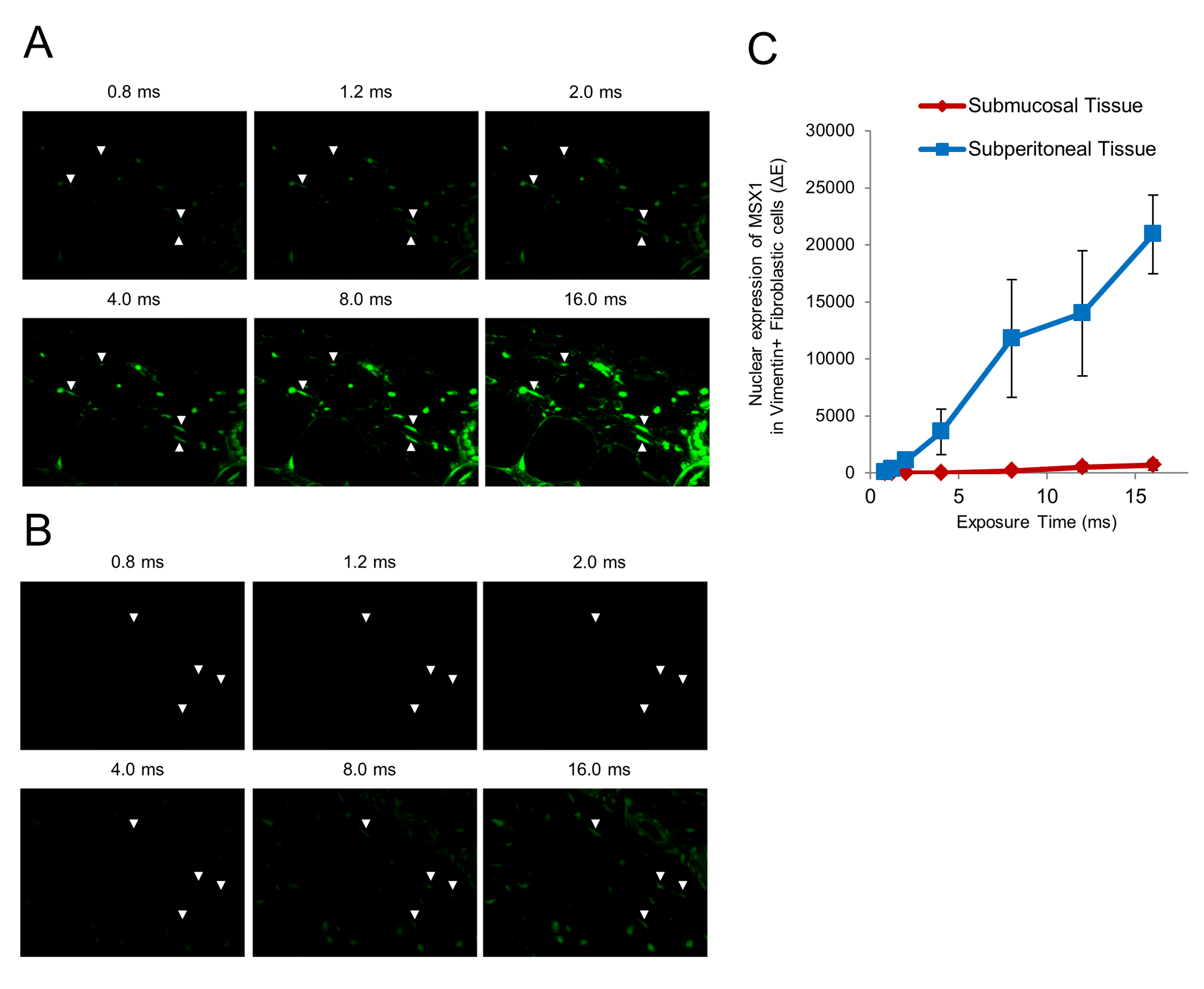

Supplement: S6 Fig — (Related to Fig 6). (A) Immunofluorescence image of MSX1 in colon subperitoneal tissue for each exposure time. The fluorescence image of MSX1 shows clear and bright at an exposure time of 8.0 ms, without any background staining. Arrow heads indicate the vimentin-positive, spindle-shaped fibroblastic cells in the picture. (B) Immunofluorescence image of MSX1 in colon submucosal tissue for each exposure time. A weak fluorescence image can be observed at exposure time 8.0 ms. Arrow heads indicate the vimentin-positive, spindle-shaped fibroblastic cells in the picture. (C) Semi-quantitation of the nuclear expression of MSX1 in human gastrointestinal tissue fibroblasts. Nuclear color difference of the vimentin-positive, spindle-shaped fibroblastic cells in each picture were measured. The error bars show the mean ± SD of 4 fibroblastic cells in the picture. (TIF) [file pone.0129241.s006.tif]
